# Mobility-Aware Privacy-Preserving Mobile Crowdsourcing †

**DOI:** 10.3390/s21072474

**Published:** 2021-04-02

**Authors:** Guoying Qiu, Yulong Shen, Ke Cheng, Lingtong Liu, Shuiguang Zeng

**Affiliations:** 1 Shaanxi Key Laboratory of Network and System Security, School of Computer Science and Technology, Xidian University, Xi’an 710071, China; gyqiu@stu.xidian.edu.cn (G.Q.); kechengstu@gmail.com (K.C.); xviviliu@gmail.com (L.L.); zengshuiguang@gmail.com (S.Z.); 2 College of Computer and Cyber Security, Hebei Normal University, Shijiazhuang 050000, China

**Keywords:** location-based service, mobile crowdsourcing application, privacy preservation, trajectory prediction, spatiotemporal markov

## Abstract

The increasing popularity of smartphones and location-based service (LBS) has brought us a new experience of mobile crowdsourcing marked by the characteristics of network-interconnection and information-sharing. However, these mobile crowdsourcing applications suffer from various inferential attacks based on mobile behavioral factors, such as location semantic, spatiotemporal correlation, etc. Unfortunately, most of the existing techniques protect the participant’s location-privacy according to actual trajectories. Once the protection fails, data leakage will directly threaten the participant’s location-related private information. It open the issue of participating in mobile crowdsourcing service without actual locations. In this paper, we propose a mobility-aware trajectory-prediction solution, TMarkov, for achieving privacy-preserving mobile crowdsourcing. Specifically, we introduce a time-partitioning concept into the Markov model to overcome its traditional limitations. A new transfer model is constructed to record the mobile user’s time-varying behavioral patterns. Then, an unbiased estimation is conducted according to Gibbs Sampling method, because of the data incompleteness. Finally, we have the TMarkov model which characterizes the participant’s dynamic mobile behaviors. With TMarkov in place, a mobility-aware spatiotemporal trajectory is predicted for the mobile user to participate in the crowdsourcing application. Extensive experiments with real-world dataset demonstrate that TMarkov well balances the trade-off between privacy preservation and data usability.

## 1. Introduction

The mobile Internet has promoted an era of “internet of everything”, bringing people a new modern life marked by information interconnection. With the help of information-sharing, mobile crowdsourcing has increasingly become a kind of popular solution for large-scale real-time missions [1]. Crowdsourcing platforms spring up everywhere all over the world. There are three parties involved in a mobile crowdsourcing application, the task publisher, platform, and participant (also named mobile user). The task publisher releases location-related tasks on the platform and provides certain rewards. Then, the platform recruits mobile users and assigns crowdsourcing tasks. Finally, the mobile users complete the assigned tasks and obtain the corresponding rewards. As a new type of data perception and service model, mobile crowdsourcing provides massive low-cost high-flexibility multi-source data. According to these data, crowdsourcing platforms provide various mobile services. Mobile crowdsourcing has been widely implemented in many fields, including environmental monitoring, treatment, intelligent transportation, social services, etc.

Privacy preservation and the service’s quality is a contradiction in mobile crowdsourcing applications. We consider the platform is honest but curious. It assigns crowdsourcing tasks according to the mobile user’s locations. Therefore, it wants to obtain the user’s location information as accurately as possible, so as to improve its service’s quality [2,3]. Even worse, the events of data leakage and various inferential attacks make the mobile user’s location-related privacy face increasing threats [4,5]. The adversaries, even the crowdsourcing platform, can analyze the mobile user’s behavioral patterns according to mobile trajectories and further infer location-related sensitive information [6]. According to these whereabouts information, customized advertisements, or other personalized services may be recommended to the mobile user without permission. It may even result in serious threats to the safety of the user’s life and property.

In recent years, the location privacy-preservation in mobile applications has attracted wide interests in both academic and industrial worlds, and many privacy-preserving techniques have been proposed. Privacy information retrieval (PIR) performs corresponding encryption and decryption operations to protect private data [7]. However, these cryptographic algorithms bring expensive computational overhead. In the literature [8,9], different kinds of location-obfuscation techniques, such as generalization and perturbation have been proposed. Location-generalization methods generalize the exact location into a certain area, and the perturbation techniques add random noise to the actual location. These methods protect the mobile user’s location-privacy with the uncertain probabilistic models. Differential privacy (DP) provides rigorous privacy-preservation [10]. Jin et al. sensed the free spectrum in the form of crowdsourcing based on the DP principle [11]. However, the DP methods are generally customized for specific application scenarios. Worst of all, all of these techniques achieve privacy preservation based on the user’s actual locations. Once their protections fail, the user’s location-related sensitive information will face direct threats.

In present privacy-preserving mobile applications, the mobile user always participates in the applications with fake positions generated by the privacy-preserving techniques, hiding their actual locations. We can regard this process as the hidden Markov model (HMM), taking the shared fake locations as the observation sequence, and the actual trajectory as the hidden-state sequence [10,11]. Therefore, HMM-based algorithms are often used to model the mobile user’s behavioral patterns. For example, the Viterbi algorithm can be used to infer the user’s maximum possible transfer path [12], and the forward-backward algorithm may reveal where the user is most likely to appear at a certain moment [13].

**Our ideas.** We analyze and model the mobile user’s behavioral patterns according to the HMM principle and further predict a mobility-aware trajectory that the user is most likely to travel. For achieving the privacy-preserving mobile crowdsourcing, the mobile user participates in the application with the predicted trajectory instead of the actual one. However, there are still huge challenges in achieving our target, mainly reflected in the following aspects.

- **Challenge 1.** The traditional Markov method can only model the steady-state transitions. However, mobile user often travels with different patterns at different times in the real world. Therefore, to model the user’s dynamic mobility based on the Markov chain, the first thing to overcome is the time correlation that the traditional Markov model lacks, in both the transfer model and steady-state distribution [14,15];
- **Challenge 2.** Considering the incompleteness of sampling data, how to apply the random sampling methods flexibly for establishing an unbiased spatiotemporal Markov model needs to be fully taken into consideration;
- **Challenge 3.** Performance-evaluation issues, such as how many POIs are generated in each time partition is suitable, and how much the generated POIs are related to the user, need to be evaluated with appropriate indicators.

Contributions. Facing the above challenges, we have made the following contributions during the process of mobility modeling and trajectory prediction.

- We have sorted out the existing location privacy-preserving techniques, analyzed their technical vulnerabilities, and finally clarified our research problem;
- A time-partitioning concept has been introduced into the traditional Markov model, forming a new spatiotemporal Markov, named TMarkov. TMarkov can model the mobile user’s time-varying behavioral patterns;
- We have performed an unbiased estimate of the TMarkov model, according to the Gibbs Sampling method;
- We have selected suitable technical indicators carefully and conducted extensive experiments with the real-world dataset to evaluate the performance of TMarkov.

## 2. Problem Formalization

This section presents a general application system, discusses the adversary model, and describes our design goals to clarify our research problem further. Table 1 summarizes some important symbols for convenience. We refer to x[i] as the *i*th element of *x* and *E* as the expectation.

### 2.1. General Privacy-Preserving Mobile Crowdsourcing

**Figure 1:**
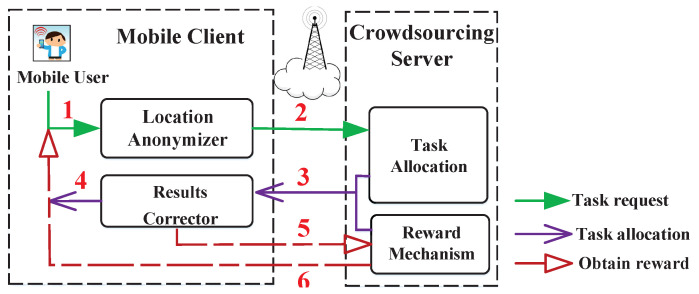
A general privacy-preserving crowdsourcing scenario.

Participating in location-based crowdsourcing threatens the mobile user’s location privacy, as analyzed in Section 1 previously. Therefore, a privacy-preserving system is widely needed in various crowdsourcing applications. Here, we present a general architecture of the privacy-preserving crowdsourcing in Figure 1. To protect location privacy, the mobile user sends the crowdsourcing platform a generated trajectory to participate in the task assignment. Afterwards, they correct the reward’s deviation caused by privacy protection, according to the actual locations. We describe its detailed dataflow as follows.

Dataflow. (1) The mobile user sends a location-related query; (2) the anonymizer protects the query’s position by generating a fake location, and sends it to the crowdsourcing platform; (3) the server responds to the query with crowdsourcing tasks and corresponding reward; (4) the result corrector corrects the response’s deviation; (5) the accepted task and the reward’s deviation is fed back to platform; and (6) the mobile user obtains the corresponding reward.

### 2.2. Attack Models

**Figure 2:**
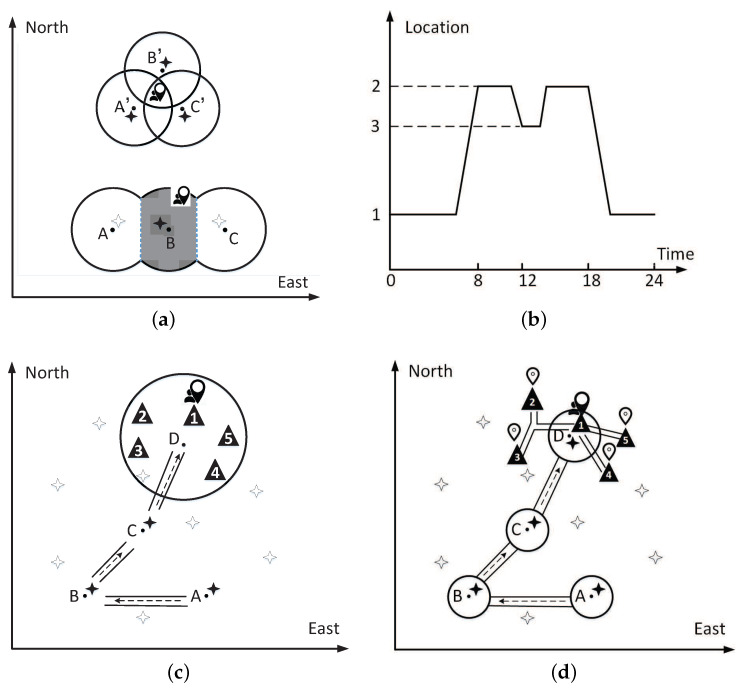
Location/trajectory inferential attacks in crowdsourcing scenarios. (**a**) Inferential attacks based on crowdsourcing elements. (**b**) Semantic analysis of trajectory. (**c**) Inferential attacks based on continually shared locations. (**d**) Road network constraints.

During the process of mobile crowdsourcing, the attacker may be the platform and other adversaries. We assume the platform is honest but curious. In order to improve the quality of its service, it seeks to infer the user’s location information as accurately as possible. The platform has a more powerful attack-capability than other adversaries because it masters the user’s historical mobile data. Therefore, this paper takes the platform as the major defense-object. Figure 2 presents the regular inferential attacks where the platform often launches.

- **Inferential attacks based on crowdsourcing elements.** According to the task’s location accepted by the user and the predesigned maximum acceptable distance (MAD), the platform can draw a circular area, taking the accepted task as the center and MAD as the radius. The circle is the effective area that the user must be in when doing the crowdsourcing task, as shown in Figure 2a. The above subfigure shows the scenario when the user accepts three tasks simultaneously. The user must appear in the intersection of the three effective areas. The bottom subfigure presents the area inference when the user accepts task *B* but rejects tasks *A* and *C*. The user must be in the area close to *B* but away from *A* and *C*;
- **Semantic analysis on the mobile trajectories.** Traveling trajectory is the information carrier of user’s daily mobile semantics. The correlated information between the locations and the mobile user may review the user’s sensitive privacy. In Figure 2b, we take a trajectory along which a user travels on a normal working day as an example. A user leaves position 1 in the morning, stays at position 2 in the morning and afternoon, and returns to position 1 in the evening. It is easy to infer that the location 1 is the user’s home and location 2 is the place where the user works;
- **Inferential attacks based on continually shared locations.** The platform has accumulated a large number of historical trajectories. It can analyze the spatiotemporal correlations hidden in the mobile data, model the user’s mobility, and then may infer the user’s following behavior. As shown in Figure 2c, after the user has completed tasks A,B,C, the platform may be able to infer that location 1 is the place where the user is most likely to visit next, according to the spatiotemporal-correlation inferential attacks;
- **Inferential attacks based on road-constraints and other background-knowledge.** In practical applications, the platform may re-identify the user-generated locations based on the real-world’s road-network constraints. As shown in Figure 2d, only position 1 is actually reachable in the effective area corresponding to task *D*. Other background knowledge may also be used in this way by the platform, such as the user’s social relationships.

### 2.3. Our Design Goals

Our ultimate design-goal is to defend against the inferential attacks which may be launched by the platform and achieve the privacy-preserving crowdsourcing applications. Here, we analyze the detailed design-goals of this paper.

Among the above four types of inferential attacks, the first two are more intuitive and easier to defend. However, because of the spatiotemporal-correlation in the user’s mobility, the latter two are more subtle and difficult to handle. Therefore, we need to propose a privacy-preserving solution based on the spatiotemporal-correlation modeling. In the solution, the actual location must be protected by the anonymous positions where the user is most likely to visit. At the same time, the anonymous positions must meet the regular semantics of the user’s mobility and be within the effective area of the accepted task. In this way, we can fully resist the platform’s inferential attacks and achieve the privacy-preserving crowdsourced applications effectively.

## 3. System Model

To achieve privacy-preserving mobile crowdsourcing, we propose our TMarkov solution. It fully considers the attack capacity that the adversary may have, and predicts the trajectory that the user is most likely to travel along. A mobile user can replace and protect their actual trajectory with the predicted trajectory. In this section, we describe TMarkov’s system model and discuss its design rationality.

### 3.1. A Glimpse of TMarkov’s Application Scenario

**Figure 3:**
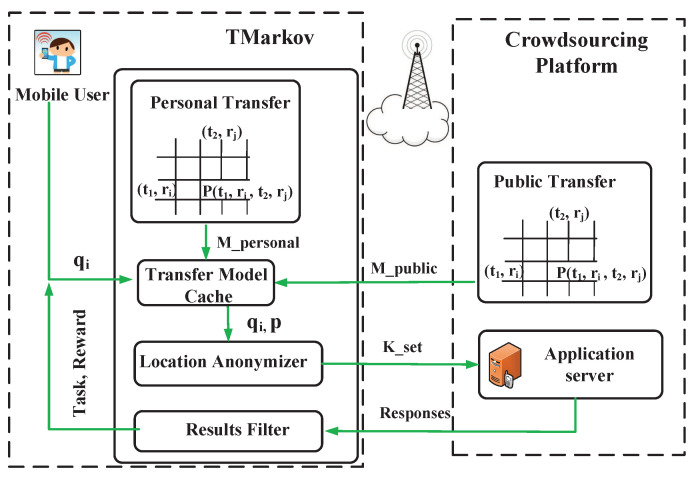
A glimpse of TMarkov’s application.

We present TMarkov’s application scenario to clarify its functional positioning further. TMarkov plays the role as the location anonymizer in the general privacy-preserving crowdsourcing as shown in Figure 1 and Figure 3, which presents a glimpse of our TMarkov’s application. Before the user participates in the application, we first generate a Personal Transfer model on the local client, or generate a Public Transfer model based on public mobile data on the platform, and download it to the Transfer Model Cache. By starting the app, the user sends a task request, $q_{i}$. Then, the Transfer Model generates the user’s time-related probability distribution, *p*, and sends $q_{i},p$ together to the Location Anonymizer. The K-anonymous set is constructed by the Anonymizer and is sent to the platform server. After receiving the request, the Server responds to the locations in the anonymous set one by one. Finally, the Results Filter filters the server’s responses and returns the exact result (task and reward) to the user.

### 3.2. System Design and Its Rationality

**Figure 4:**
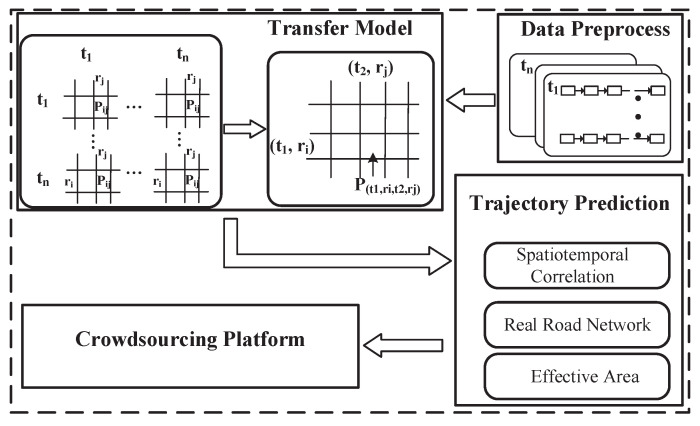
System model.

As described above, TMarkov needs to complete two functions, modeling the user’s mobility and predicting a mobility-aware trajectory that the user is most likely to travel along. Therefore, we have designed three system components as shown in Figure 4, building a new transfer model, unbiased training, and trajectory prediction. In the following, we introduce each component separately and analyze the rationality of its design.

Modeling Section. The traditional Markov chain can only model the user’s steady-state transition because there is only one probability value between each two-state-pair item. However, mobile user’s behavioral patterns change over time. For example, the locations the mobile user visits on weekends are different from the places corresponding to weekdays. Therefore, we introduce the time-partitioning concept into traditional Markov chain, building a time-related Markov model to record the user’s time-varying mobility. It overcomes the limitations of traditional Markov on dynamic mobility modeling.

Model Training. The mobile data obtained by either the platform or the local client is incomplete. Therefore, we need to select a suitable random-sampling method to estimate the unbiased mobile model.

Behavioral Trajectory Prediction. During the prediction process, we need to fully take the attacker’s capacity into consideration for defending against the potential inferential attacks effectively. Specifically, the actual location is protected by an anonymity set constructed by the locations that the user is most likely to visit, according to the above mobility modeling. The anonymous locations are selected with the rules that need to meet the real road-network constraints and be within the accepted task’s effective area.

## 4. Mobility-Aware Trajectory Prediction

In this section, we describe the functional components of the main results specifically, including: Time-related mobility perception, the model’s unbiased estimation, future behavioral-trajectory prediction, and the solution’s complexity analysis.

### 4.1. Time-Related Mobility Perception

**Figure 5:**
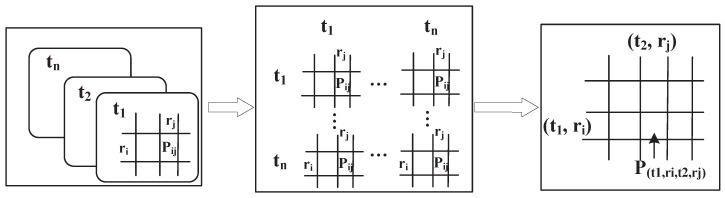
A new transfer model. A time-partitioning concept is introduced into the traditional Markov chain for recording mobile user’s time-varying behavioral patterns.

As mentioned earlier, the Markov transition matrix can only record the spatial transition model in the short-term stable transition state [16]. It has nothing to do with the initial state and it cannot record the user’s time-dependent mobile patterns [17]. In response to these limitations of Markov, our solution, TMarkov, introduces the time factor into the Markov spatial transition probability matrix in the form of Time Partitioning, forming a new transfer model as shown in Figure 5. It can record and perceive the mobile user’s time-varying dynamic behavioral patterns.

To build the new transfer model, we introduce the time-partitioning concept from $LPM^{2}$ [18,19] into the Markov transition matrix, for perceiving the mobile user’s time-varying dynamic behavioral patterns. As shown in Figure 5, we first divide the target time-period into time partitions in the data processing stage. For example, the period from 8 am to 6 pm is divided into 10 partitions. Then, we match the mobile data into corresponding time partitions, and aggregate them into a super-matrix based on the spatiotemporal transfers within and between time partitions. The state space of the super-matrix becomes a combination of time and location, (T,S), adding a time-prefix *T* to the location-state item *S*. It can record not only the spatial transfer of the events but also the occurrence’s time. *T* represents the time-partition which means the period the event happens. Its accuracy can be adjusted flexibly. *S* indicates the location set in the target area.

We control the accuracy of the user’s mobility perception by configuring T,S. For example, we can divide a week into weekdays and weekend, or separate a day into three parts, morning, afternoon, and evening. Or taking a part of one day into consideration, such as the working hours from 8 a.m. to 6 p.m. In addition to configuring the scopes of T,S, we can also control their division’s precision, dividing them into small units with the scales of n,m respectively. A finer division means that more mobile events will be recorded separately, a stronger spatiotemporal correlation can be modeled, and finally indicates the user’s mobility will be perceived more accurately by our transfer model.

### 4.2. The Transfer Model’s Unbiased Estimation

Based on the transfer model proposed above, we can construct the TMarkov mobile model for perceiving the mobile user’s dynamic behaviors. We give a brief description of the basic principles of Markov theory.

**Lemma** **1.** *Detailed Balance Conditions. If the transfer probability P and the distribution π of the periodic Markov chain satisfy:*

(1) $$\pi \left(i\right)*P_{ij}=\pi \left(j\right)*P_{ji}\;for\;all\;i,j$$

*where π is the steady distribution of the Markov chain, and the above equation is called Detailed Balance Condition.*

In vector form:

(2) $$\sum_{i=1}^{\infty }\pi \left(i\right)*P_{ij}=\sum_{i=1}^{\infty }\pi \left(j\right)*P_{ji}=\pi \left(j\right)$$

that is:

πM=π.

We construct the TMarkov transition matrix based on the above transfer model and train it with user’s personal historical trajectories data.

Due to the incompleteness of the sampling data, in most cases, the transfer matrix *M* we constructed does not satisfy with the balance condition. The traditional approach to this problem is to introduce an acceptance rate to balance two sides of the equation:

(3) p(i)p(i,j)α(i,j)=p(j)p(j,i)α(j,i).

Obviously, since the acceptance rate is a decimal number, its sampling efficiency is low. MCMC, Metropolis–Hastings, and Gibbs Sample all seek to find an acceptance rate equal or close to 1. In this paper, we adopt the Gibbs Sampling method, as it can solve the random sampling problem in this paper with a two-dimensional sampling.

**Figure 6:**
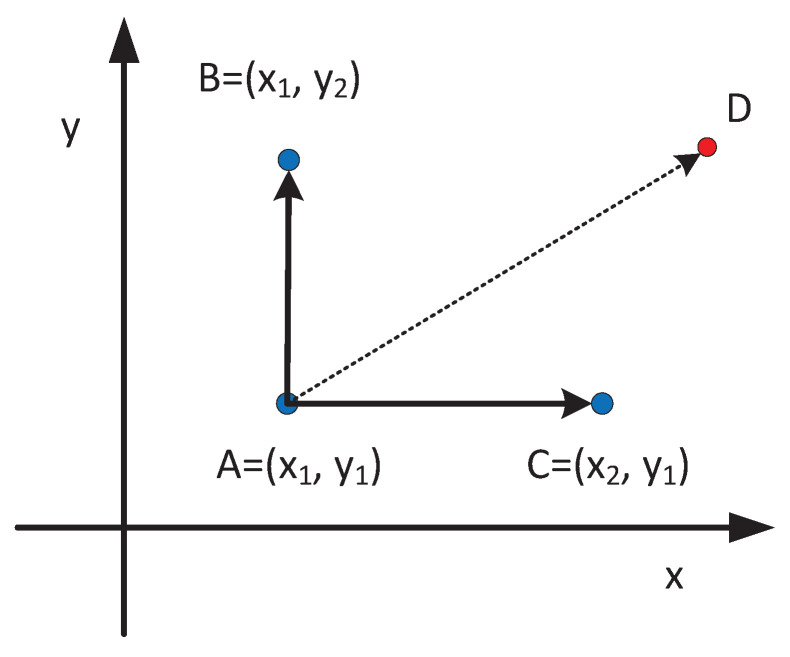
Transferable two points.

**Gibbs Sampling.** Give an example of Gibbs Sampling’s two-dimensional sampling. For the joint probability distribution P(x,y), and two points $A(x_{1},y_{1})$, $B(x_{1},y_{2})$ as shown in Figure 6. We can easily find:

$$\left\{\begin{array}{cc} & p\left(x_{1},y_{1}\right)p\left(y_{2}|x_{1}\right)=p\left(x_{1}\right)p\left(y_{1}|x_{1}\right)p\left(y_{2}|x_{1}\right)\\ & p\left(x_{1},y_{2}\right)p\left(y_{1}|x_{1}\right)=p\left(x_{1}\right)p\left(y_{2}|x_{1}\right)p\left(y_{1}|x_{1}\right) \end{array}\right.$$

that is:

(4) $$p\left(A\right)p\left(y_{2}|x_{1}\right)=p\left(B\right)p\left(y_{1}|x_{1}\right).$$

As shown in Figure 6, we can perform the probable transfer p(y∣x) separately in one-dimensional space each time, and finally achieve the transition between *A* and *D* in two times. The balance condition is satisfied during this transition process. Specifically, we get universal Gibbs Sampling as Algorithm 1.

**Algorithm 1:** Two-dimensional Gibbs sampling      1 Initialization $X_{0}=x_{0}\;Y_{0}=y_{0}$      2 At *t* = 0,1,..., loop sampling            2.1 $y_{t+1}∼p\left(y|x_{t}\right)$            2.2 $x_{t+1}∼p\left(x|y_{t+1}\right)$

In this paper, we refer to TT as the real training trajectory set, and ET as the hypothetical full dataset. Then, they have the following probability relation:

(5) $$P\left(M|TT\right)=\sum_{ET}P\left(M,ET|TT\right).$$

However, it is unfeasible to sample from Pr(M,ET∣TT) directly. Its computation will increase exponentially with the length of the user’s mobile trajectory. Therefore, we estimate the missing data by making use of the Gibbs Sampling to unbiased estimate the new transfer model, achieving the user’s mobility perception effectively.

The whole process can be completed in polynomial time. A complete iteration of the Gibbs method yields the following sample pairs $\left(M^{l},ET^{l}\right)$, sampling from two conditional distributions separately:

(6) $$M^{l}∼P\left(M|ET^{l-1},TT\right)$$

(7) $$ET^{l}∼P\left(ET|EM^{l},TT\right).$$

From the TMarkov transition matrix M, we sample independently by row, considering Dirichlet as its prior distribution. Then, the $M_{i}$’s lth sampling can be described as follows:

(8) $$Dirichlet{\left[Cnt_{ij}\left(ET^{l-1}\right)+\epsilon_{ij}\right]}_{j=1...M}$$

where Cnt means the number of transitions, ϵ represent the mobility constraints.

To sample from ET, we simplify the method in the literature [19] and perform the lth sampling as follows:

(9) $$\frac{P_{ET(t-1)ET(t)}^{l}b\left(TT(t)|ET(t)\right)P_{ET(t)ET(t+1)}^{l}}{\sum_{r\in R}P_{ET(t-1)r}^{l}b\left(TT(t)|r\right)P_{rET(t+1)}^{l}}.$$

The values $P_{ET(0)ET(1)}^{l}$ and $P_{ET(T)ET(T+1)}^{l}$ are defined to be 1. The function $b\left(r|ET(t)\right),r\in TT$ is equal to be 0, if r≠∅ and r≠ET(t). Otherwise, it is equal to 1. $b(r_{i}|r_{j})$ is the probability for the situation that $r_{i}$ is reported as $r_{j}$. We take *n* samples, T=1,...,t.

In order to construct a stable TMarkov transfer matrix, we can preset the iteration’s number for the Gibbs Sampling operation, or use the approximation between the last-two samplings as a condition for the end of the iteration.

### 4.3. Future Behavioral Trajectory Prediction

Similar to the traditional Markov theory, we can easily obtain the user’s time-related steady-state distribution and spatiotemporal transition matrix from the TMarkov transfer model.

First of all, to predict and protect the user’s initial behavior, we take the user’s probability distribution on-location set from the steady-state distribution in the corresponding time partition and arrange it in descending order. Finally, top n locations (top n POIs, points of interest) are picked up as an anonymity set to protect the location the user visits initially.

Secondly, to predict and protect the subsequent behaviors. Above all, we need to get the corresponding time partition when the user participates in the crowdsourced application. Then, taking the position closest to the task accepted previously as the basic point, we transform and obtain the user’s probability distribution on-location set, according to the TMarkov transfer matrix. Finally, the same operations are performed as in the previous situation. Top n POIs are picked up from the probability distribution, forming the anonymity set to predict and protect the user’s actual behavior.

During the construction of the above anonymity set, we also need to make the anonymous locations fully meet the road-network constraints in the real world and be within the accepted task’s effective area. Considering that the user accepts the task near their actual location, we replace the accepted task’s effective area with the user’s maximum acceptable area at the actual location (i.e., acceptable area). We choose the top n POIs within the acceptable area corresponding to the actual position.

### 4.4. Complexity Analysis

**Theorem** **1.** *During the implementation process, our TMarkov takes $O\left(nmd\right)$ time, n,m,d are the numbers of time-partitions, divided geographic grids, and iteratives of Gibbs sampling, respectively.*

**Proof.**  The cost of executing TMarkov is mainly spent on the transfer model’s training, denoted as T(TMakov). □

Due to the sampling data’s incompleteness, we train the TMarkov transfer model by implementing the Gibbs Sampling method in two dimensions, i.e., the target matrix *M* and the estimated completion ET. Both of their matrices’ scales are (nm)×(nm) and we produce the samples for each row separately. Therefore, this operation needs to be executed 2 nmd times, i.e.,T(STMakov)∼O(nmd).

## 5. Experimental Evaluation

In this section, we analyze and evaluate the performance of TMarkov by conducting extensive experiments on real-world datasets. During the experiment process, we achieve the mobility modeling part in C++. Other modules are implemented in MATLAB on a PC with 2.60 GHz*2 Intel i7 CPU and 16 GB memory (HP, Palo Alto, CA, USA).

### 5.1. Real-World Dataset

We adopt the real-world dataset from the Geolife project (Microsoft Research Asia) [20,21,22]. This GPS trajectory dataset is contributed by 182 users during a period of over five years (from April 2007 to August 2012), containing 17,621 trajectories with a total distance of 1,292,951 km and a total duration of 50,176 h. A total of 91.5% of the trajectories are logged in a dense representation, e.g., every 1∼5 s or every 5∼10 m per point.

**Figure 7:**
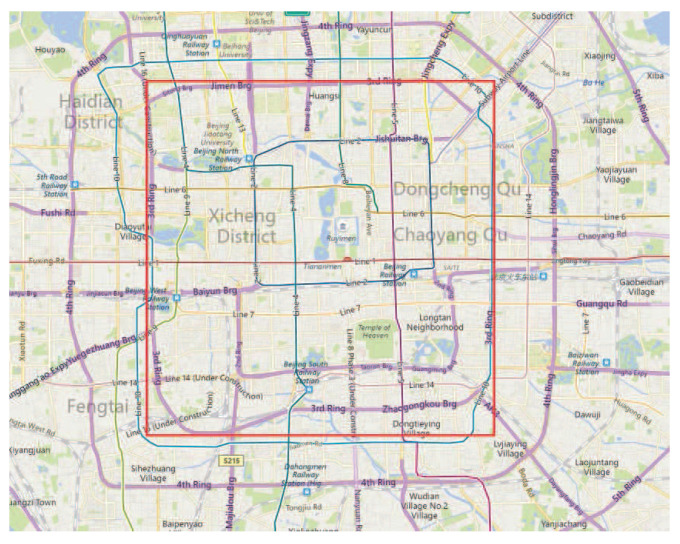
The target area of the 3rd Ring-Road of Beijing.

**Figure 8:**
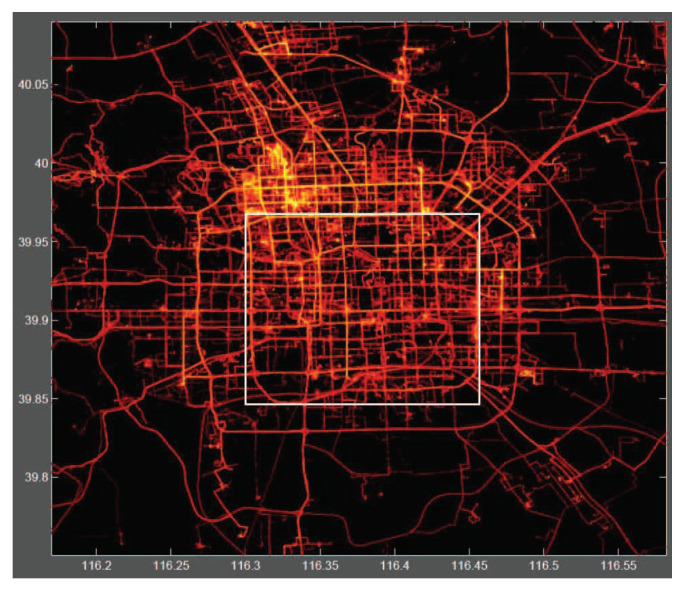
The data distribution’s heat map within the 5th Ring-Road of Beijing.

Although this dataset is wildly distributed in over 30 cities of China and even in some cities located in the USA and Europe, the majority is created in Beijing, China. We chose the area within the 3rd Ring-Road of Beijing as the target area, as shown in Figure 7, taking the data distributed within the target area for our experiments. Figure 8 shows the trajectory distribution’s heat map within the 5th Ring-Road of Beijing. From the heat map, it can be seen that our selected area locates at the core of the trajectory distribution, which ensures there are sufficient trajectories for mobility analysis. Our TMarkov conducts mobility modeling based on personal trajectory data. Therefore, we selected the mobile data of the top 10 users in descending order based on the number of personal trajectories for experimental analysis. Table 2 presents the statistical data of the dataset actually used.

### 5.2. Experimental Setting

This paper aims to predict the mobile user’s future traveling trajectories by modeling mobile behaviors. A mobile user participates in the application with the locations most likely to visit to protect their actual trajectory, achieving the privacy-preserving mobile crowdsourcing.

We conduct extensive experiments in a general crowdsourcing scenario, taking a normal weekday as the target prediction period. Assuming that the platform releases crowdsourcing tasks in each hour, we first divide the prediction period into *t* time partitions. Then, according to TMarkov, a predicted trajectory is constructed based on the top *n* POIs derived out from the user’s probability distribution in each time partition. In the experiments, we take t=10 and n=1∼5 for constructing the predicted trajectory. It means that the prediction period is divided into 10 time partitions, and we take 1∼5 high-frequency visited locations from each time partition.

We also calculate out the top *n* places that the user most visits in each time partition, based on the statistic methods. These locations are used to form a statistical trajectory as the baseline to compare with the predicted trajectory.

For the geographic representation, we consider an area of 12.8×12.8 [km × km] within the Third-Ring Road of Beijing as illustrated in Figure 7 and divide the area into a 20×20 grids of equal size. We repeat the entire experiment 20 times, taking their statistical results as the experimental performance presentation.

### 5.3. Predicted Trajectory Exhibitions

**Figure 9:**
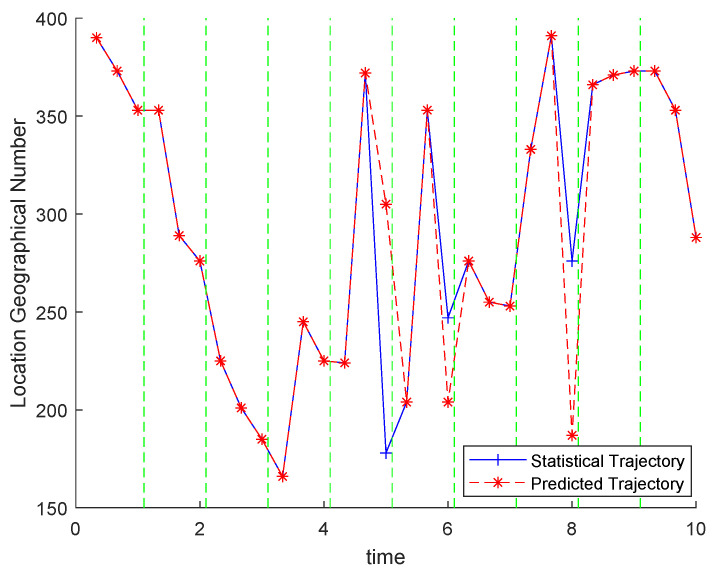
Random sample exhibition of the top 3 temporal POIs sequence.

To understand the experimental results intuitively, we directly present a predicted trajectory randomly chosen from the experimental results. Among the 20 groups of results, we took the top 3 locations with the highest frequency of occurrence in each time partition as the top 3 POIs, forming a top 3 POIs sequence over the entire time period as the predicted trajectory. Then, we calculate out the corresponding top-3-POIs statistical trajectory and present the two trajectories as shown in Figure 9.

Figure 9 presents the (time, geographic number) pair sequence of the two trajectories. We mark the locations in trajectory with geographic numbers. Among the 30 (time, location) pairs in the two trajectories, only two pairs are different from each other. Observing the time information of these two pairs, we find that they correspond to the hours at noon and after users leave work in the afternoon. In real life, the user’s travel in these two periods has greater uncertainty. Such as where to eat lunch at noon, and where to relax after leaving work in the afternoon. Overall, the presentation shows that our mobility modeling solution and its behavioral prediction results are effective.

### 5.4. Average Coverage Ratio

**Figure 10:**
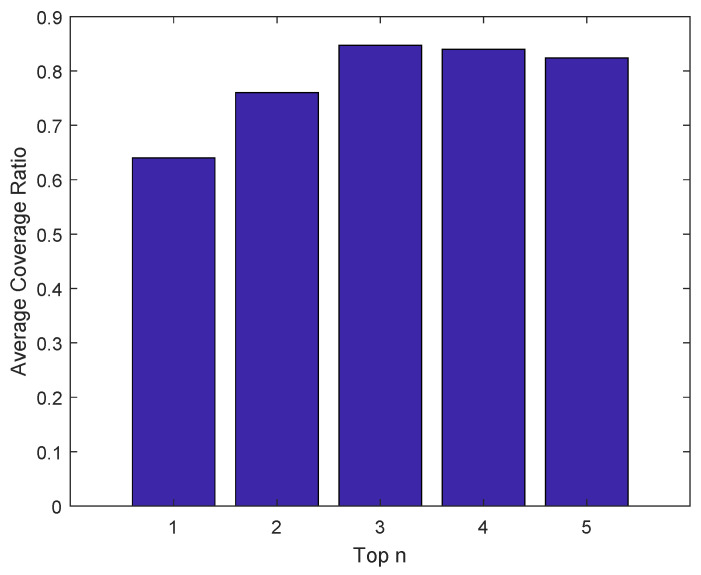
Average coverage ratio of the predicted trajectories, the top n POI sequences.

The average coverage ratio refers to the proportion of the POIs shared by the two tracks, taking the statistical trajectory as the basis of fact. Therefore, the predicted trajectory’s coverage rate relative to the statistical track can be referred to as a performance indicator of the prediction’s accuracy. To verify TMarkov’s prediction performance thoroughly, we set *n* values from 1 to 5, respectively. Figure 10 shows the average coverage of the predicted trajectories.

As shown in Figure 10, when the top 1 POI is taken from each corresponding time partition, the coverage ratio of the predicted trajectories is relatively low, only 64%. This is due to the fact that mobile user often has more diverse behavior patterns in some time partitions, such as at noon and in the period after getting off work in the afternoon. During these periods, there are more alternative locations for the user to choose. When top 3 POIs are taken, the average ratio of our TMarkov’s predicted results reaches the highest value, up to 84.7%. This is because that more frequently visited locations are selected into the predicted trajectories, as the number of POIs chosen from each time-partition increases. Therefore, TMarkov’s prediction accuracy obtains a significant improvement. However, when we take the top 5 POIs, the coverage ratio becomes dropping, due to the introduction of low-frequency POI. When too many POIs need to be introduced from corresponding time partitions, a few low-frequency POIs will be selected into the predicted trajectory from the periods when user’s behavior pattern is relatively single.

Therefore, for serving the crowdsourced application best with TMarkov, we recommend extracting top 3 POIs from each time partition, taking this top 3 POI sequence as the predicted trajectory.

### 5.5. Cumulative Distribution of Time-Partition’s Proportion on the Coverage Ratio

In the previous subsection, we showed the overall accuracy of the top *n* POI sequence predicted by TMarkov. Here, we analyze the proportion distribution of the time partitions on the coverage ratio according to TMarkov’s predicted results. That is the proportion of the time partitions when the accuracy of TMarkov’s prediction reaches the corresponding coverage rate.

**Figure 11:**
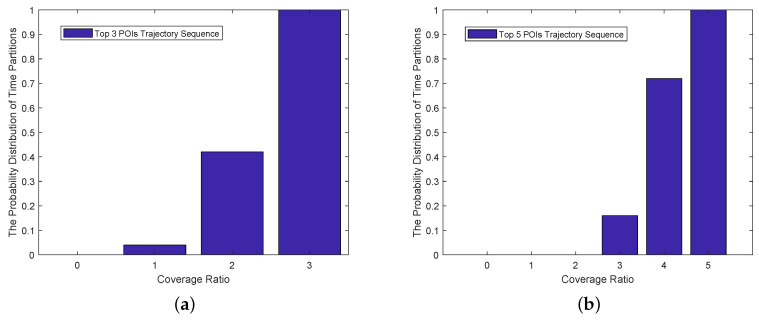
Cumulative distribution of the time-partition’s proportion on the coverage ratio. (**a**) Top 3 temporal POIs sequence. (**b**) Top 5 temporal POIs sequence.

We express the cumulative distribution of time-partition’s proportion on the coverage ratio as $P(Cover\left(tp_{ij}\right)\leq m)$, where $Cover(tp_{ij})$ denotes the coverage rate of the *i*th user in the *j*th time partition and *m* is the corresponding coverage value. We show this cumulative distribution in Figure 11. It presents the proportion distribution of the time partitions on the coverage ratio, clearly.

In the predicted top-3-POI trajectory, the proportion of time partitions who have a coverage of Two is 38%, and the proportion of the ones with full coverage (Three) is 58%. That is, up to 96% of the time partitions with coverage greater than Two. In the top-5-POI trajectory, coverage rate Four corresponds to 56% of the time partitions, and Five corresponds to 28%. It means that up to 84% of the time partitions with coverage greater than Four.

Figure 11 shows that, when n takes the value of 3, more than 2 high-frequency POIs predicted in 96% of the time partitions are shared with the statistical trajectory in the predicted top-3-POI sequence. When n takes 5, more than 4 high-frequency POIs predicted in 84% of the time partitions are shared with the statistical ones in the predicted top-5-POI sequence. Two groups of experimental results indicate that TMarkov has high practical availability.

## 6. Discussion

### 6.1. Application Modes of Our TMarkov

We discuss our TMarkov’s application modes and their privacy-preserving performances in the following three scenarios.

- Deploy our TMarkov solution in the online application. The platform already has plenty of the user’s historical mobile data. It can model the spatiotemporal correlations hidden in the user’s mobility and launch the inferential attacks to infer the user’s actual travel. Our design goal is to resist the platform’s inferential attacks while minimizing the changes to the existing architecture and facilities.
  TMarkov models the spatiotemporal association based on the user’s personal historical data to simulate the platform’s attack-capacity. An anonymous set is constructed using the user’s most likely locations to visit within a certain area. Our TMarkov breaks the spatiotemporal correlation that the platform’s attacks rely on, realizing the privacy-preserving mobile application;
- The second scenario corresponds to the cold-start problem of new user in online applications. The platform does not have any of the user’s personal historical data. In this scenario, the platform does not have the capacity to infer and attack the new user’s personal behavior patterns.
  To prevent the platform from obtaining the new user’s traveling information, we can replace personal mobile data (personal data sets) with public users’ traveling data (public datasets). According to the TMarkov trained with public datasets, the location’s popularity can be characterized based on public users’ access. Before participating in the application, the user pre-specifies a small area (or area-sequence) based on the actual travel. TMarkov builds an anonymous set consisting of the most popular locations within the specified area. The platform recommends crowdsourcing tasks to the new user.
  Here, the attacker can only infer the user’s travel based on the crowdsourcing task accepted by the user. A circle can be drawn as the effective area where the user must visit while doing the task, taking the accepted task’s position as the center, and the user’s maximum traveling distance for accepting the task as the radius. The user usually takes 5 km as the maximum acceptable distance. Then, the effective area of the platform’s attack is a circular area with a diameter of 10 km. This area is quite large that we think the user’s location privacy is safe in this case;
- The third scenario is the platform that is newly launched without any data. Here, the platform has the weakest attack capability. The users only need to adopt the spatial privacy-preserving method to protect their actual locations. Such as generalization (generalize the location into a small area), obfuscation (build an anonymous set with surrounding POIs), perturbance (add random noise to the actual position based on the DP principle), etc.

### 6.2. Future Work

We further discuss the optimization space of our TMarkov and take the following ideas as the research directions of our future work.

- The space complexity of our TMarkov is relatively high. In the experiments, we divided the area within Beijing’s 3rd Ring-Road into 20 × 20 equal-size blocks, and the target time-period from 8 a.m. to 6 p.m. into 10 time-partitions. Finally, our TMarkov has the transfer matrix with dimensions as high as 4000 × 4000.
  Observing the trained TMarkov transfer matrix, we find that it is very sparse, and most of the transition probability is 0. It is because the number of locations visited in the general-user’s daily life is very limited. Based on this discovery, we can optimize its modeling space with the points of interest that the user actually visited and stayed. This optimization can significantly reduce the space complexity of TMarkov;
- Our TMarkov has a strong universality, but its function is relatively single. We can consider further developing and designing new privacy-preserving mechanisms based on it. TMarkov generates the user’s time-related probability distribution on location-set and models the user’s time-varying mobile behavior patterns. Therefore, it can be widely applied in mobile modeling scenarios. Taking it as a core component for mobile modeling, we can further develop and design new privacy-preserving mechanisms for solving more complex security issues.
  For example, we can update the user’s time-related probability distribution between two participations in a continual crowdsourcing scenario based on the Bayesian posterior theorem, taking the accepted task as a condition. Then, we continue to execute TMarkov for the user to participate in the crowdsourcing application again. Such an improved solution can eliminate the privacy risk brought by the accepted tasks on the user’s subsequent participation. We can also provide the differential privacy (DP) mechanism with the anonymous set constructed from the user’s time-related probability distribution generated by TMarkov, for achieving the spatiotemporally correlated DP solution. And so on.

## 7. Related Work

In this section, we review some prior works that are most relevant to our TMarkov. We compare these works from two aspects, the spatiotemporal mobility modeling and the privacy preservation in crowdsourcing scenario.

**Spatiotemporal mobility modeling.** Modeling the user’s mobile behaviors is an open issue in crowdsourcing applications [16,23]. Literatures [17,24] modeled user’s mobile behaviors based on the Markov method. However, they failed to overcome the limitations of traditional Markov. The PLP solution [25] takes the temporal correlation into consideration, according to the conditional random fields. However, it depends on specific scenarios and is difficult to popularize. Our TMarkov not only models the spatial transitions, but also records their time correlation.

**Privacy-preservation in crowdsourcing applications.** Existing location privacy-preserving techniques [26] provide anonymous or uncertain privacy-preservation generally for mobile applications, such as the obfuscation [27], generalization [28,29], perturbation [30], etc. However, their protections are vulnerable to the inferential attacks due to the user’s mobility [12,31].

Solutions, such as θ-secure area [32], DPSence [11], and PLP [25], introduced the spatiotemporal correlation into the location privacy-preservation. θ-secure area [32] assessed whether the clustering area was secure, by comparing the Earth mover’s distance between the prior and posterior distributions. It relies more on statistical calculations and does not dig the spatiotemporal mobility sufficiently. DPSence [11] provided a crowdsourced spectrum-sensing solution with the DP principle based on the Markov model. However, the spatial transfer, it modeled, did not consider the temporal correlation. Literature [25] proposed the PLP solution to model the continual transfer according to conditional random fields (CRF), while the CRF method has poor compatibility with other privacy-preserving mechanisms.

Our TMarkov solution models the user’s behavioral patterns and protects the user’s actual position with the locations most likely to visit. The spatiotemporal correlation in the user’s mobility has been thoroughly considered into the privacy-preservation. While protecting the user’s mobile privacy effectively, it also has a wide range of universality to various mobile scenarios.

## 8. Conclusions

Targeting a privacy-preserving crowdsourcing application, we proposed a mobility-aware trajectory prediction solution, TMarkov, for hiding and protecting the user’s actual travel. According to Markov Chain, it introduced a new time-partitioning concept to model the mobile user’s time-varying behavioral patterns. Extensive experiments with real-world data demonstrated that TMarkov could predict the user’s traveling trajectory accurately, with an average accuracy rate of nearly 85%. Among them, it covered almost all of the high-frequency visited locations in 96% of the time partitions. TMarkov generated a mobility-aware predicted trajectory based on the user’s most likely locations to visit for the mobile user to participate in crowdsourcing, instead of the actual traveling trace. TMarkov protects the user’s mobile privacy effectively while ensuring the application’s smooth progress. 

## Figures and Tables

**Table 1:** Parameter settings.

Parameter	Setting
π	Markov steady distribution
*M*	Markov transfer matrix
$p_{i}$ or p(i)	Probability distribution of state *i*
$p_{i,j}$ or p(i,j)	Transition probability from state *i* to state *j*
*T*	Time partitions set
*t*	Timestamps
*S*	Location set
x,y,z	Locations

**Table 2:** Statistics of the aataset actually adopted.

Indicators	Mean	Standard Deviation
Trajectory Length (GPS locations)	2134	380.850
Trajectory Time-span (hours)	4.6	1.088
The Number of Trajectories per User	157.8	17.210
The Time-span of Trajectories per User (months)	6.2	1.166

## Data Availability

Data sharing not applicable.

## References

[1] Chen Y., Lv P., Guo D., Zhou T., Xu M. (2018). Task and participant matching in mobile crowd sensing: A Survey. Springer J. Comput. Sci. Technol..

[2] Chen Y., Guo D., Lv P., Zhou T., Xu M. ProSC: Profit-driven participant selection in compressive mobile crowdsensing. Proceedings of the 26th International Symposium on Quality of Service (IWQoS).

[3] Chen Y., Lv P., Guo D., Zhou T., Xu M. (2017). Trajectory segment selection with limited budget in mobile crowd sensing. Elsevier J. Pervasive Mob. Comput..

[4] Gramaglia M., Fiore M., Tarable A. Preserving mobile subscriber privacy in open datasets of spatiotemporal trajectories. Proceedings of the IEEE INFOCOM 2017—IEEE Conference on Computer Communications.

[5] Oya S., Troncoso C., Perezgonzalez F. Back to the drawing board: Revisiting the design of optimal location privacy-preserving mechanisms. Proceedings of the 2017 ACM SIGSAC Conference on Computer and Communications Security.

[6] Gotz M., Nathn S., Gehrke J. Maskit Privately releasing user context streams for personalized mobile applications. Proceedings of the 2012 ACM SIGMOD International Conference on Management of Data.

[7] Papadopoulos S., Bakiras S., Papadias D. (2010). Nearest neighbor search with strong location privacy. Springer VLDB Endow..

[8] Andres M.E., Bordenabe N., Chatzikokolakis K., Palamidessi C. Geo-indistinguishability: Differential privacy for location-based systems. Proceedings of the 2013 ACM Sigsac Conference on Computer & Communications Security.

[9] Gedik B., Liu L. (2008). Protecting location privacy with personalized k-anonymity: Architecture and algorithms. IEEE Trans. Mob. Comput..

[10] Xiao Y., Xiong L. (2014). Protecting locations with differential privacy under temporal correlations. arXiv.

[11] Jin X., Zhang R., Chen Y. DPSense: Differentially private crowdsourced spectrum sensing. Proceedings of the 2016 ACM SIGSAC Conference on Computer and Communications.

[12] Shokri R., Theodorakopoulos G., Boudec J.L., Hubaux J. Quantifying location privacy. Proceedings of the 32nd IEEE Symposium on Security and Privacy, S&P 2011.

[13] Shokri R., Theodorakopoulos G., Troncoso C. Protecting location privacy: Optimal strategy against localization attacks. Proceedings of the 2012 ACM Conference on Computer and Communications Security.

[14] Lv Q., Mei Z., Qiao Y., Zhong Y., Lei Z. Hidden markov model based user mobility analysis in LTE network. Proceedings of the 2014 International Symposium on Wireless Personal Multimedia Communications (WPMC).

[15] Li X., Lian D., Xie X., Sun G. Lifting the predictability of human mobility on activity trajectories. Proceedings of the 2015 IEEE International Conference on Data Mining Workshop (ICDMW).

[16] Bhakti M., Shelar D., Chitre D.K. (2015). Hidden markov model with biclustering cache replacement policy for location based services in MANET. Int. J. Eng. Comput. Sci..

[17] Guo B., Liu Y., Wang L., Li V.O., Lam J.C., Yu Z. (2018). Task allocation in spatial crowdsourcing current state and future directions. IEEE Internet Things J..

[18] Shokri R., Theodorakopoulos G. (2011). Location Privacy Meter Tool. Location Privacy. https://github.com/rzshokri/quantifying.

[19] Robert C., Celeux G., Diebolt J. (1993). Bayesian estimation of hidden Markov chains: A stochastic implementation. IEEE Stat. Probab. Lett..

[20] Zheng Y., Zhang L., Xie X., Ma W. Mining interesting locations and travel sequences from GPS trajectories. Proceedings of the ACM WWW.

[21] Zheng Y., Li Q., Chen Y., Xie X., Ma W. Understanding mobility based on GPS Data. Proceedings of the ACM Ubicomp.

[22] Zheng Y., Xie X., Ma W. (2010). GeoLife: A collaborative social networking service among User, location and trajectory. IEEE Data Eng. Bull..

[23] Ouyang K., Shokri R., Rosenblum D. A non-parametric generative model for human trajectories. Proceedings of the IJCAI.

[24] Jiang J., Pan C., Liu H., Yang G. Predicting Human Mobility Based on Location Data Modeled by Markov Chains. Proceedings of the IEEE UPINLBS.

[25] Ma Q., Zhang S., Zhu T. (2016). PLP: Protecting location privacy against correlation analyze attack in crowdsensing. IEEE Trans. Mob. Comput..

[26] Ghinita G. (2013). Privacy for Location-Based Services. Synth. Lect. Inf. Secur. Privacy Trust..

[27] Nergiz M., Atzori M., Saygin Y., Guc B. (2009). Towards Trajectory Anonymization: A Generalization-Based Approach. Trans. Data Priv..

[28] Chen R., Fung B., Desai B.C., Sossou N. Differentially private transit data publication: A case study on the Montreal transportation system. Proceedings of the ACM KDD.

[29] Krumm J. (2009). A survey of computational location privacy. Pers. Ubiquitous Comput..

[30] Poulis G., Skiadopoulos S., Loukides G., Gkoulalas-Divanis A. (2014). Apriori-based algorithms for k^m^-anonymizing trajectory data. Trans. Data Priv..

[31] Shokri R., Stronati M., Song C. Membership inference attacks against machine learning models. Proceedings of the IEEE SP.

[32] Lee B., Oh J., Yu H., Kim J. Protecting location privacy using location semantics. Proceedings of the ACM SIGKDD.

